# Rapid increases in obesity in Jamaica, compared to Nigeria and the United States

**DOI:** 10.1186/1471-2458-8-133

**Published:** 2008-04-23

**Authors:** Ramón A Durazo-Arvizu, Amy Luke, Richard S Cooper, Guichan Cao, Lara Dugas, Adebowale Adeyemo, Michael Boyne, Terrence Forrester

**Affiliations:** 1 Department of Preventive Medicine and Epidemiology, Loyola University Medical School, Maywood, IL, USA; 2 National Human Genome Center, Howard University, Washington, DC, USA; 3 Tropical Medicine Research Institute, University of the West Indies, Kingston, Jamaica

## Abstract

**Background:**

Weight gain in adulthood is now common in many populations, ranging from modest gains in developing countries to a substantial percentage of body weight in some Western societies. To examine the rate of change across the spectrum of low to high-income countries we compared rates of weight change in samples drawn from three countries, Nigeria, Jamaica and the United States.

**Methods:**

Population samples from Nigeria (n = 1,242), Jamaica (n = 1,409), and the US (n = 809) were selected during the period 1995–1999 in adults over the age of 19; participation rates in the original survey were 96%, 60%, and 60%, respectively. Weight in (kg) was measured on 3 different occasions, ending in 2005. Multi-level regression models were used to estimate weight change over time and pattern-mixture models were applied to assess the potential effect of missing data on estimates of the model parameters.

**Results:**

The unadjusted weight gain rate (standard error) was 0.34(0.06), 1.26(0.12), 0.34(0.19) kg/year among men and 0.43(0.06), 1.28(0.10), 0.40(0.15) kg/year among women in Nigeria, Jamaica, US, respectively. Regression-adjusted weight change rates were significantly different across country, sex, and baseline BMI. Adjusted weight gain in Nigeria, Jamaica and US was 0.31(0.05), 1.37(.04), and 0.52(0.05) kg/year respectively. Women in Nigeria and the US had higher weight gains than men, with the converse observed among Jamaicans. The obese experienced weight loss across all three samples, whereas the normal weight (BMI < 25) had significant weight gains. Missing data patterns had an effect on the rates of weight change.

**Conclusion:**

Weight change in sample cohorts from a middle-income country was greater than in cohorts from either of the low- or high-income countries. The steep trajectory of weight gain in Jamaica, relative to Nigeria and the US, is most likely attributable to the accelerating effects of the cultural and behavioral shifts which have come to bear on transitional societies.

## Background

The effect of overweight and obesity on health outcomes, including cardiovascular disease [1-6], asthma and other respiratory diseases [7-10], and diabetes [11-15] have been extensively documented. Relative weight began to rise sharply in the U.S. in the mid-1980's and the "obesity epidemic" has begun to receive widespread attention [16-21]. Although heterogeneity in obesity prevalence's currently exists among U.S. sub-populations, the rate of change among adults is virtually identical across social class, gender and race [22-31]. The mechanism underlying the upward trend in relative weight is assumed to be rooted in macro-social changes in consumption and associated behaviors, however there is relatively little empirical research relating change in socio-cultural patterns to age-related weight gain.

Overweight and obesity prevalence rates have also been increasing in many low and middle income countries [32-35]. In an international comparative study of 85 countries mean levels of body mass index (BMI) and other cardiovascular (CV) risk factors were found to have an asymmetric up side down U-shaped association with a country's stage of development, whereby mean BMI levels were highest in middle income countries [32]. The absolute rate of change on the "ascending limb" – moving from low to middle income status – appears to be more rapid than the corresponding rate associated with the transition from middle to high income. These data suggest that a critical "take off" point is being reached in middle income countries, where widespread adoption of consumer behaviors fuels the epidemic of CV risk factors. Given the rapid cultural shifts taking place in these countries it may be possible to observe the associated social processes more directly.

Cross-sectional analyses of obesity prevalence reflect the accumulated history of calorie excess but are relatively insensitive to the dynamic processes that may reflect recent changes in obesity-promoting exposures. The rate of change in weight in a population may be a better predictor of future risk. We therefore undertook a cross-cultural comparison of rate of change in weight in a series of cohorts recruited using comparable sampling methods and examination protocols.

The Word Bank classifies economies for analytical and operational purposes according to the gross national income (GNI) per capita. Countries with a GNI of $US 735 or less in 2002 were termed low-income, whereas a middle-income economy corresponded to GNI less than $US 9,076, but more than $US 735. High-income economies are comprised by those countries with GNI per capita of $9,076 or more. According to the World Bank, Nigeria had a GNI per capita of $US 300, Jamaica $US 2,690 and the United States $US 35,400 [36]. The countries involved spanned the range of economic development, representing Africa, the Caribbean and North America.

## Methods

### Study populations

An analytical data set was put together for these analyses from our studies conducted in Jamaica, US (Maywood, Il) and Nigeria during the period 1994–1999 and resulted in a final data set consisting of a total of 3, 460 participants of which 1,409 were from Jamaica, 809 were from the US and 1,242 individuals were from Nigeria. Participants included in these analyses had at least two weight measurements. Participation rate at baseline varied from 60% in the United States to 61% in Jamaica and 95% in Nigeria. The average time between weight determinations for subjects with three measurements was 3.8 years (N = 612), 2.2 years (N = 468), and 0.9 years (N = 396) for Jamaica, Nigeria and the United States, respectively. A brief description of the data collection for each country is presented below:

#### Jamaican Data

Jamaican participants were recruited into the International Collaborative Study of Hypertension in Blacks (ICSHIB) project. ICSHIB was undertaken to describe the distribution of blood pressures, hypertension prevalence and risk factors among seven populations of West African origin [37,38]. In Jamaica, participants were recruited from Spanish Town, a working class suburb of Kingston. Recruitment was based on the probability proportional to size sampling method in which appropriate geographic subunits were identified in each community (enumeration areas in Jamaica) and a random sample was generated, proportional to the size of the subunit [37,39]. Data collected included anthropometrics (weight, height, body mass index, waist and hip circumference, and waist/hip ratio), blood pressure and prevalence of hypertension. Fieldwork was begun in 1994 and, to date, three cycles of study exams have been completed (baseline and 2 follow-up exams). The study protocol was reviewed and approved by the institutional review board at the University of the West Indies, Mona, Jamaica.

#### Nigerian data

Participants were recruited into the Genetic Study of Hypertension in Blacks between 1995–1999.

Participants were recruited from the rural villages of Igbo-Ora and Idere in southwestern Nigeria, Oyo State. The sampling frame for this study was provided by ICSHIB, as described earlier. After identification of appropriate geographical subunits a random sample was generated proportional to the size of the subunits. Middle aged probands (aged 25–54 years) were identified and invited to participate along with all available first degree relatives over the age of 12 [40-42]. Over the next 7 years, participants were asked to return for measurements of blood pressure, weight and body composition, resulting in between one and three follow-up examinations.

This sample was supplemented by unrelated individuals enrolled through a separate study monitoring weight change [39]. The study protocols were reviewed and approved by the institutional review board at the University of Ibadan in Nigeria.

#### US data

Participants from the US were recruited into the Genetic Study of Hypertension in Blacks between 1995–1999 and the Fat Reduction Intervention Trial in African-Americans (FRITAA) between 1995–1998.

The Fat Reduction Intervention Trial in African-Americans (FRITAA) was a 5-year randomized controlled trial evaluating a community intervention with the goal of reducing fat and cholesterol intake in Maywood, Illinois [43,44]. Households were designated as the unit of intervention and were contacted through local media, house-to-house canvassing and telephone calls. Four hundred and twenty households were recruited and assigned to either active intervention or observation for 18 months. Weight was measured at baseline and on 2 other separate occasions for the duration of the study. As a result of average weight not differing across intervention groups, the two arms of the study were combined. The study protocol was reviewed and approved by the institutional review board at Loyola University Medical Center in the US.

### Statistical methods

Data are presented as means (standard deviations), and percentages. Analysis of variance (ANOVA) was used to compare the means of all continuous variables (e.g. body mass index, weight, height, etc) measured at baseline across the three countries. The chi-squared statistic was used to test baseline distributions of categorical variables. These statistical techniques were also used to test for potential effect of missing data pattern by comparing baseline characteristics for those subjects with incomplete data (only two weight determinations) and those who had all three weight measurements within each country-sex subgroup. Unadjusted weight change was defined as the last minus the baseline weight measurement divided by the time elapsed between determinations. A p-value level of p < 0.05 was accepted for statistical significance.

Multi-level regression, also termed mixed-effect, random coefficient, hierarchical, growth curve models [45-47] were fit to the data to examine the association between weight change and time accounting for other covariates, such as sex, country, baseline age and body mass index. These methods allow for comparisons of weight change by site and sex, and for modeling individual patterns of weight change. Furthermore, rates of weight change at different body mass index can be estimated and compared to investigate the effect modification of baseline BMI on weight change.

An initial model including terms to assess whether age modified the association between weight and time was fit, but interaction terms were subsequently dropped due to lack of statistical significance. On the other hand, sex by time was kept in the final model to test for differences in weight change among men and women within country. Baseline BMI is assumed to have an effect not only on the mean weight, but also on the rate of weight change over time. We reflected this observation by adding BMI, represented by 3 categories, by time interaction terms to the statistical model fit. In the final model age adjustment was carried out by including age at baseline centered at the country-sex specific mean.

In addition, we apply random-effects pattern-mixture models [48] to examine the influence of missing data patterns on weight change estimates. Briefly, subjects are divided into groups corresponding to their missing data pattern and then the multi-level analysis proposed above applied to each group, resulting in a rate of weight change for each missing data pattern. A combined estimate of the rate of change and the corresponding standard errors can be obtained as suggested by Hogan and Laird [49]. Specifically, the data were analyzed for completers, subjects with all three weight determinations, and for non-completers (individuals who were lost to follow-up after the second visit). To assess the effect of missing data on the rate of change over time and across country, interaction terms of the pattern indicator variable, COMP, with time and country were included in a multi-level regression model. Analyses were carried out using the statistical package STATA, version 10.0 [50].

## Results

### Descriptive

Baseline characteristics by site and sex are presented in Table 1, for completers (all three weight determinations) and non-completers (baseline and one follow-up weight measurement) and for the two groups combined. The study sample represents 1,242 participants from Nigeria (577 men, 665 women), 1,409 from Jamaica (556 men, 853 women) and 809 from the US (214 men, 595 women) with at least one weight assessment after baseline. The average(standard deviation) weight gain was 0.39(1.49) kg/year (men: 0.34(1.39) kg/year, women: 0.43(1.56) kg/year) in Nigeria, 1.27(2.92) kg/year (men: 1.26(2.77) kg/year, women: 1.28(3.02) kg/year) in Jamaica, and 0.38(3.50) kg/year (men: 0.34(2.83) kg/year, women: 0.40(3.71) kg/year) in the United States. As expected, baseline average BMI's were greater in the US than in Jamaica and Nigeria. The prevalences of overweight (obesity) were 81% (51%), 54% (24%), and 20% (5%) in the US, Jamaica and Nigeria, respectively.

**Table 1:** Baseline characteristics by sex and country. Baseline characteristics for all the subjects retained for analysis and lost to follow-up by country and sex.

	**Sample Size^†^**	**Age (sd)**	**Weight^‡ ^(sd^§^)**	**Height^# ^(sd)**	**BMI^†† ^(sd)**	**Overweight (bmi ≥ 25)**	**Obesity (bmi ≥ 30)**	**Weight Change^‡‡ ^(sd)**
***All subjects***								
**Nigeria**	1,242	47(16.8)	59(11.9)	163(8.5)	22.2(4.2)	20%	5%	0.39(1.49)
*Women*	665	46(16.1)	58(12.8)	159(6.9)	23.0(4.8)	27%	8%	0.43(1.56)
*Men*	577	48(17.4)	61(10.6)	169(6.7)	21.3(3.2)	12%	1%	0.34(1.39)
								
**Jamaica**	1,409	46(13.8)	72(16.1)	165(8.6)	26.5(6.1)	54%	24%	1.27(2.92)
*Women*	853	46(13.4)	73(17.4)	161(6.4)	28.2(6.5)	67%	34%	1.28(3.02)
*Men*	556	46(14.4)	71(13.7)	172(6.8)	23.9(4.3)	34%	9%	1.26(2.77)
								
**US**	809	47(13.8)	88(21.2)	167(8.9)	31.5(7.4)	81%	51%	0.38(3.50)
*Women*	595	46(14.1)	87(21.3)	163(6.6)	32.4(7.6)	84%	56%	0.40(3.71)
*Men*	214	47(12.9)	91(20.8)	177(7.1)	29.2(6.4)	74%	39%	0.34(2.83)

***Completers***								
**Nigeria**	468	50(15.1)	60(12.0)	164(8.4)	22.3(4.2)	20%	6%	0.52(1.20)
*Women*	221	48(14.8)	58(12.8)	158(6.5)	23.2(4.8)	29%	10%	0.61(1.19)
*Men*	247	51(15.2)	61(11.0)	169(6.5)	21.4(3.4)	13%	2%	0.43(1.21)
								
**Jamaica**	612	48(13.6)	72(16.5)	165(8.2)	26.8(6.3)	55%	26%	1.65(3.33)
*Women*	392	47(13.5)	73(17.9)	161(6.3)	28.3(6.6)	67%	34%	1.60(3.55)
*Men*	220	48(13.6)	71(13.5)	172(6.3)	23.9(4.4)	33%	11%	1.74(2.92)
								
**US**	396	46(13.7)	88(20.7)	166(8.7)	31.8(7.1)	84%	55%	0.28(4.06)
*Women*	308	45(13.9)	86(20.3)	164(6.8)	32.2(7.2)	85%	58%	0.21(4.30)
*Men*	88	49(12.5)	95(21.1)	176(7.1)	30.4(6.6)	82%	45%	0.51(3.08)

***Non-completers***								
**Nigeria**	774	45(17.4)	59(11.8)	163(8.6)	26.3(6.0)	20%	5%	0.31(1.63)
*Women*	344	44(16.5)	58(12.7)	159(7.0)	23.0(4.8)	26%	8%	0.34(1.71)
*Men*	330	46(18.6)	60(10.2)	169(6.9)	21.1(3.1)	11%	1%	0.28(1.51)
								
**Jamaica**	797	45(13.9)	72(15.8)	166(8.8)	26.3(6.0)	53%	23%	0.98(2.53)
*Women*	461	45(13.2)	73(17.0)	161(6.5)	28.1(6.4)	67%	34%	1.00(2.45)
*Men*	336	45(14.8)	71(13.9)	172(7.2)	23.9(4.3)	34%	7%	0.94(2.63)
								
**US**	413	47(13.9)	88(21.7)	168(9.1)	31.3(7.7)	79%	48%	0.48(2.85)
*Women*	287	47(14.3)	87(22.3)	164(6.3)	32.5(8.1)	83%	54%	0.60(2.53)
*Men*	126	46(13.1)	89(20.3)	177(7.2)	28.4(6.1)	69%	34%	0.22(2.64)

Weight change per year from baseline was also estimated as last weight determination minus weight at baseline divided by the elapsed time between the first and last weight measurements. Data were collected from 1995 to 1999 from Nigeria, Jamaica and the United States (US).

* p < 0.05, ** p < 0.01. ^†^Number of subjects at baseline. ^‡^Weight in Kilograms. ^§^sd = standard deviation. ^#^Height in centimeters.

^††^BMI = Body Mass Index in kg/m^2^. ^‡‡^Weight change in kilograms per year.

**Figure 1:**
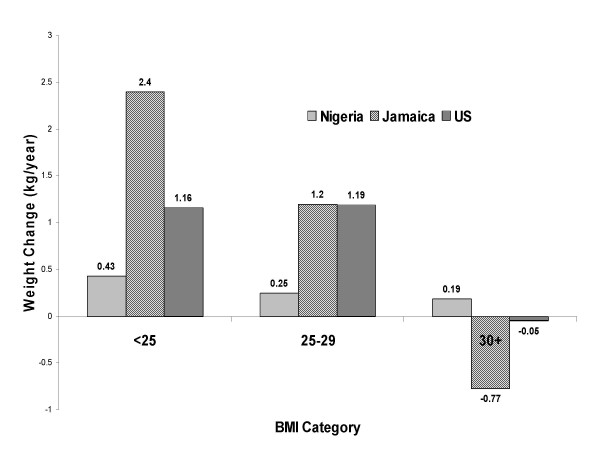
**Estimated weight change by country and BMI group**. Unadjusted weight change (last weight determination minus baseline weight divided by time between measurements) by country, Nigeria, Jamaica, and the US for each of three BMI categories (BMI < 25, 25 <= BMI < 30, BMI >= 30). Data were collected from 1995 to 1999 from Nigeria, Jamaica and the United States (US). Only participants with at least two weight determinations are depicted in this figure.

An illustration of the effect of baseline BMI on weight change is depicted in Figure 1, where the unadjusted weight change estimates are plotted against BMI category for each of the three countries.

**Figure 2:**
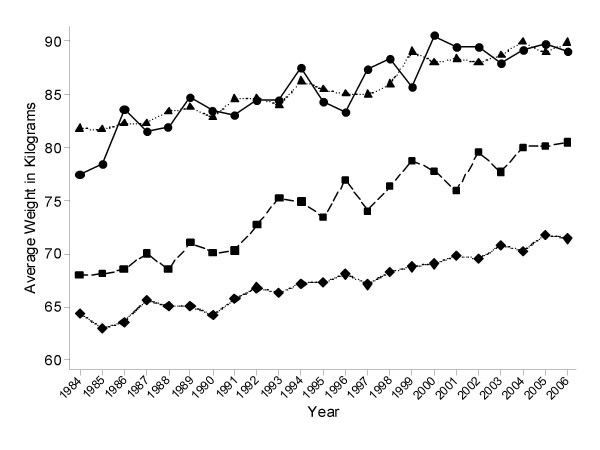
**Trends in weight in Illinois, USA by sex from 1984 to 2006**. Age-adjusted average weight in kilograms per year for each of four sex-ethnic groups in Illinois, United States. Data were collected between 1984 and 2006 for all adults 20–75 years of age, as part of the Behavioral Risk Factors Surveillance System (BRFSS). ● Black Males, ▲ White Males, ■ Black Females, ◆ White Females. Estimated rate of weight change (standard errors) among these groups are: 0.47(0.05), 0.38 (0.01), 0.60(0.04), and 0.37 (0.02) for Black Males, White Males, Black Females and White Females, respectively.

The longitudinal analyses presented here are potentially biased if persons who are more likely to lose weight were differentially lost to follow-up. Comparisons of baseline characteristics of persons who did not complete the study and those with complete information at all three visits are depicted in Table 1. There were no significant differences for most of the characteristics, except for age, which was significantly different among US women and Nigerian men. There were statistical differences observed in average weight and prevalence of overweight among Jamaican men, however, these differences are small and may reflect the effect of large sample sizes. External validation from cross-sectional surveys conducted over the same time period was only possible in the US. Population average weights in four sex-race/ethnic groups in Illinois in the 1984–2006 Behavioral Risk Factors Surveillance System (BRFSS) are displayed in Figure 2. The change in average weight per year was 0.54 kg/year among blacks (men: 0.47 kg/year; women 0.60 kg/year), and 0.37 kg/year among whites (men: 0.38 kg/year; women 0.37 kg/year). Therefore these estimates closely resemble those obtained in the US sample used in this report.

### Model-Based

The models selected incorporated variables representing sex, country, baseline age (centered at country-sex specific mean value), baseline BMI, time of weight measurement from baseline, and sex-time, BMI-time interaction terms. The sex-time interaction was statistically significant (p < 0.001), indicating different rates of weight change among males and females within a country. Similarly, the two-way interaction time, and country with the pattern indicator variable, COMP, were statistically significant, suggesting an effect of the missing data pattern on the rate of weight change. The final model did not include three-way interactions due to the lack of statistical significance of these interactions, which were tested via the likelihood ratio test.

#### Completers versus non-completers

Weight gain among completers was greater than non-completers for all three countries. These results can be extracted from Tables 2 and 3. For instance, among completers from Nigeria, Jamaica, and the US the weight gain (standard error) was 0.51(0.07), 1.58(0.05), and 0.65(0.08) kg/year respectively, whereas the corresponding values for non-completers were 0.19(0.07), 1.21(0.05) and 0.40(0.07) kg/year.

**Table 2:** Model-based estimates of weight change for non-completers by sex, country and BMI group.

	**Body Mass Index (BMI) Groups**							
	**BMI < 25**		**25 ≤ BMI < 30**		**BMI ≥ 30**		**Combined**	
				
	N*	**Δ^† ^weight (SE^‡^)**	**N**	**Δ weight (SE)**	**N**	**Δ weight (SE)**	**N**	**Δ weight (SE)**
**NON-COMPLETERS**								
**Nigeria**	623	0.48(0.09)	113	-0.57(0.12)	38	-2.25(0.15)	774	0.19(0.07)
*Women*	330	0.78(0.13)	80	-0.38(0.15)	34	-2.18(0.16)	444	0.34(0.10)
*Men*	293	0.14(0.12)	33	-1.02(0.16)	4	-2.82(0.18)	330	-0.01(0.11)
								
**Jamaica**	372	2.13(0.08)	245	1.11(0.09)	180	-0.53(0.11)	797	1.21(0.05)
*Women*	151	2.51(0.11)	153	1.35(0.12)	157	-0.45(0.12)	461	1.11(0.07)
*Men*	221	1.87(0.11)	92	0.71(0.13)	23	-1.09(0.15)	336	1.35(0.08)
								
**US**	87	2.08(0.11)	129	0.99(0.11)	197	-0.73(0.11)	413	0.40(0.07)
*Women*	48	2.37(0.16)	85	1.21(0.15)	154	-0.59(0.13)	287	0.43(0.09)
*Men*	39	1.73(0.15)	44	0.57(0.16)	43	-1.23(0.16)	126	0.31(0.09)

Estimated rate of weight change per year by multi-level regression model. Data were collected from 1995 to 1999 from Nigeria, Jamaica and the United States (US). Only individuals who do not have all three weight determinations are included (non-completers).

* Sample size. ^†^Δ weight = weight change in kilograms per year. ^‡^SE = standard error.

**Table 3:** Model-based estimates of weight change for completers by sex, country and BMI group.

	**Body Mass Index (BMI) Groups**							
	**BMI < 25**		**25 ≤ BMI < 30**		**BMI ≥ 30**		**Combined**	
				
	N*	**Δ^† ^weight (SE^‡^)**	**N**	**Δ weight (SE)**	**N**	**Δ weight (SE)**	**N**	**Δ weight (SE)**
**COMPLETERS**								
**Nigeria**	373	0.81(0.09)	69	-0.24(0.11)	26	-1.88(0.14)	468	0.51(0.07)
*Women*	158	1.18(0.12)	41	0.02(0.15)	22	-1.78(0.16)	221	0.67(0.09)
*Men*	215	0.54(0.12)	28	-0.62(0.16)	4	-2.42(0.18)	247	0.36(0.10)
								
**Jamaica**	276	2.57(0.08)	179	1.58(0.09)	157	-0.16(0.10)	612	1.58(0.05)
*Women*	129	2.91(0.11)	131	1.75(0.11)	132	-0.05(0.12)	392	1.52(0.07)
*Men*	147	2.27(0.11)	48	1.11(0.13)	25	-0.69(0.15)	220	1.68(0.08)
								
**US**	63	2.60(0.14)	113	1.42(0.13)	220	-0.31(0.13)	396	0.65(0.08)
*Women*	47	2.76(0.17)	81	1.60(0.17)	180	-0.20(0.15)	308	0.73(0.10)
*Men*	16	2.13(0.17)	32	0.97(0.18)	40	-0.83(0.17)	88	0.36(0.11)

Estimated rate of weight change per year by multi-level regression model. Data were collected from 1995 to 1999 from Nigeria, Jamaica and the United States (US). Only individuals who have all three weight determinations are included (completers).

* Sample size. ^† ^Δ weight = weight change in kilograms per year. ^‡^SE = standard error.

#### All Subjects Combined

Due to the heterogeneity of parameter estimates for completers and non-completers reflected by the significance of the interaction terms constructed between time, and country with missing data pattern, combined parameter estimates and corresponding standard errors for all subjects were obtained. These estimates are depicted in Table 4. Weight change differed significantly by country, with Jamaica experiencing larger weight gains than both Nigeria and the US. Weight gain (standard error) in Jamaica (1.37(0.04) kg/year) was approximately 4 times larger than the corresponding gain in Nigeria (0.31(0.05) kg/year), and the US (0.52(0.05) kg/year). Rates of weight change are dependent on body weight at the time of baseline measurement, with those of normal weight (BMI < 25) experiencing weight gains of 0.60(0.06), 2.32(0.06), 2.30(0.09) kg/year in Nigeria, Jamaica and the US respectively. The Nigerian overweight participants (25 ≤ BMI < 30) experienced a weight loss of -0.44(0.09) kg/year compared to weight gain in overweight Jamaica and the US of 1.31(0.07) kg/year and 1.19(0.09) kg/year. Obese individuals (BMI = 30) saw their weight decline at 2.10(0.11) kg/year in Nigeria, 0.36(0.08) kg/year in Jamaica, and 0.52(0.09) kg/year in the US. Estimates by sex within country are also displayed in Table 4, where it is apparent that rates of weight change are greater for women than men.

**Table 4:** Model-based estimates of weight change for completers and non-completers by sex, country and BMI group.

	**Body Mass Index (BMI) Groups**							
	**BMI < 25**		**25 ≤ BMI < 30**		**BMI ≥ 30**		**Combined**	
				
	N*	**Δ^† ^weight (SE^‡^)**	**N**	**Δ weight (SE)**	**N**	**Δ weight (SE)**	**N**	**Δ weight (SE)**
**COMPLETERS & NON-COMPLETERS**								
**Nigeria**	996	0.60(0.06)	182	-0.44(0.09)	64	-2.10(0.11)	1,242	0.31(0.05)
*Women*	488	0.91(0.09)	121	-0.24(0.11)	56	-2.02(0.12)	665	0.45(0.07)
*Men*	508	0.31(0.09)	61	-0.83(0.12)	8	-2.62(0.15)	577	0.15(0.08)
								
**Jamaica**	648	2.32(0.06)	424	1.31(0.07)	337	-0.36(0.08)	1,409	1.37(0.04)
*Women*	280	2.69(0.08)	284	1.53(0.08)	289	-0.27(0.09)	853	1.30(0.05)
*Men*	368	2.03(0.08)	140	0.85(0.10)	48	-0.88(0.11)	556	1.48(0.06)
								
**US**	150	2.30(0.09)	243	1.19(0.09)	416	-0.52(0.09)	809	0.52(0.05)
*Women*	95	2.56(0.12)	166	1.40(0.11)	334	-0.39(0.10)	595	0.58(0.07)
*Men*	55	1.84(0.12)	76	0.73(0.12)	83	-1.04(0.12)	214	0.33(0.07)

Estimated rate of weight change per year by multi-level regression model. Data were collected from 1995 to 1999 from Nigeria, Jamaica and the United States (US). All eligible subjects are included, completers (all three weight measurements) and non-completers (only two weight determinations).

* Sample size. ^† ^Δ weight = weight change in kilograms per year. ^‡^SE = standard error.

## Discussion

Our study contrasts communities across a wide range of technological and industrial development. Longitudinal analyses of these 3 cohorts demonstrate significantly more rapid weight gain among individuals living in a middle-income country, represented by Jamaica, and confirm prior evidence based on cross-sectional prevalences. Despite the fact that Jamaican participants gained substantially more weight than did the US participants (e.g., 1.4 versus 0.5 kg/year), the prevalence of obesity was higher in the U.S., both at baseline and follow up. The prevalence of obesity was lowest in Nigeria, although during this time period participants in that rural community were gaining about as much weight as those followed in the U.S. Projected forward 5 years, Jamaicans will on average experience an increase of 2.5 BMI units, compared to 0.925 units in the U.S. sample, reaching an average BMI of 29 and 32.5 in Jamaica and the US, respectively. The accelerated weight gain among individuals of normal weight (BMI < 25) compared to weight loss in the obese (BMI >= 30) predicts a larger than anticipated increase in weight-related health problems in these countries. In particular, in Jamaica, the rate of weight gain among lean individuals is 9 times larger than the rate of weight loss among obese subjects implying a rapid right shift of the population weight distribution (2.32 kg/year versus -0.36 kg/year). This in turn will have a dramatic effect on the obesity rates and related diseases, such as diabetes and other cardiovascular conditions.

Over the last 10–15 years societies as geographically and culturally distant as Barbados, Russia, Kuwait, and Japan have all experienced rapid increases in relative weight, affecting both children and adults [51-55]. In the US the shift in the BMI trend slope occurred in the mid-1980's and a 5-fold increase in the rate of change/year has been observed subsequently [33]. Even Norway – with historically low obesity rates and high participation in leisure time physical activity – experienced an abrupt up-turn around 1990 [56-59]. Clearly some "common source exposure" is shifting the population distribution of weight right-ward and virtually all segments of the societies that participate in the world economy are being affected. While it is assumed that lifestyle changes related to the growing consumer economy are the driving force it has been difficult to define and quantify the specific factors. Consequently, examination of countries currently undergoing rapid transitions in obesity risk could shed additional light on this question.

Although obesity contributes to several major CV risk factors the greatest impact of the current increases in body weight has been on diabetes [14,60-63]. Excess body weight is the primary underlying epidemiologic measure associated with diabetes risk, however, the distribution of the diabetes epidemic is not wholly consistent with variation in BMI across populations [64]. In general, populations with recent, rapid increases in obesity, including many island nations and indigenous groups, appear to have higher than predicted diabetes rates [65-68]. For example, Jamaica is now experiencing an increasing incidence of diabetes out of proportion to mean levels of BMI [69]. If confirmed by additional surveys, this hypothesis suggests that the tempo of change in obesity risk at the population level may help explain aggregate risk. Although BMI is widely used as a proxy variable, it is an indicator variable for a complex syndrome representing several correlated lifestyle factors, including sedentarism and specific dietary patterns, among others [70-72]. It is possible that the rapid changes in obesity risk are brought about by simultaneous changes in many of these factors, unaccompanied by compensating health-promoting responses, thus leading to higher diabetes risk.

Although Jamaica has undergone rapid cultural changes over the last 20 years, accompanied by a decrease in the rural population, it has experienced stagnating or negative economic growth [73]. While accurate economic data are difficult to obtain, it is also unlikely that average Nigerians have seen any substantial increase in their material standard of living over the last 2 decades [73]. Thus, the lifestyle changes required to fuel weight gain do not require general economic development, and instead may reflect the penetration of market-based consumption patterns into stagnating or declining economies. These observations suggest that it is the character of social development, not necessarily the level of economic activity per se that is driving the combined obesity-diabetes epidemic in many poor and middle-income countries. Research on trends in nutrient intake, food preparation patterns, physical activity and other weight-modifying factors may help define the context within which these changes are taking place in countries like Jamaica.

An additional advantage of multi-level (i.e. random-effects, growth curve, hierarchical, random coefficients) models is the ability to handle data missing at random (MAR). That is, the missing values depend on the observed data, but not on the unobserved data [74,75]. It has been demonstrated by simulation studies that the proposed random effects methods yield unbiased parameter estimates under various degrees of increasing-over-time missingness [76]. However, a loss in efficiency (larger standard error) is anticipated due to the depleted information. Although it is reasonable to assume that in our study missing weight determinations are missing at random, pattern-mixture models were used to assess the effect of this assumption on the parameter estimates. When multi-level analysis was carried out on all data (completers and non-completers), thus making use of the MAR assumption, the estimates of weight change rate were within 0.1 kg/year (100 grams/year). For instance, the rate of weight change in Nigeria obtained after fitting this model was 0.40 kg/year versus 0.31 kg/year from Table 4. The corresponding estimates from Jamaica (US) are 1.40(0.42) versus 1.37(0.52) kg/year. These results support the assumption of data missing at random.

This report suffers from several limitations inherent to observational studies that need to be taken into account in the interpretation of the findings. Of course, as with all sample surveys, the inference from these to the entire population depends heavily on the assumption that these cohorts are representative. The individuals who agreed to take part in this study – and were available at follow-up – may differ in their weight trajectory from other segments of the population. Corroborating information about causal factors related to risk of weight gain were not available. Comparable measures of physical activity, nutritional patterns and obesity prevention activities were not available. Furthermore, data analysis with body mass index categorized into three groups has to be interpreted with caution due to the small number of subjects in some of the BMI groups. The extent of the problem is reduced in the aggregated estimates since the estimates are obtained using a weighted average.

The rural population of West Africa, including Nigeria, has up until this period remained remarkably lean over the life course [52]. It is therefore somewhat surprising that our longitudinal data show substantial weight gain. We have previously shown that underweight is a risk factor for mortality in this community and it is possible that differential survival biased our results.

## Conclusion

This report is consistent with the view that while increasing weight is now a global phenomenon there is substantial heterogeneity across countries in the rate of change. The annual rate of weight gain, and its first derivative, the rate of increase in the slope of weight gain, may therefore provide an additional index of population-level obesity risk that may be used for surveillance and etiologic research. Further understanding of the determinants of rapid weight change will require larger national samples from other middle-income countries with dependent economies.

## Competing interests

The authors declare that they have no competing interests.

## Authors' contributions

RDA had extensive involvement in the development of a draft and revisions of the manuscript. He performed and directed statistical analyses. AL made substantive contributions in the conception, design and planning of the studies used for this manuscript. She provided the necessary background and ideas to develop a draft and subsequent versions of the document, including the final manuscript.

RSC reviewed and edited the manuscript as necessary. He was the PI or Co-PI in the studies used for the manuscript. He contributed extensively to the discussion and final version of the manuscript. GC was involved in the management of the data and carried out statistical analysis. She participated in reviewing the manuscript and suggested changes to the data analysis provided the necessary background and ideas to develop a draft and subsequent versions of the document, including the final manuscript. LD participated in data analysis and reviewed and edited the manuscript as necessary. AA was local Co-PI for the Nigerian site and was involved in the planning and conduct of the studies. He reviewed and edited the manuscript. MB and TF were local Co-PIs for the Jamaican site. They both participated in the planning and conduct of the studies, as well as the review and editing of this manuscript. TF contributed to the conception of the manuscript along with RDA, AL and RSC.

## Pre-publication history

The pre-publication history for this paper can be accessed here:


